# Proposal of a Safe Transport Protocol and Its Utility of Antigen-Preserving Tissue for Formalin-Fixed Porcine Renal Samples

**DOI:** 10.3390/biomedicines13040831

**Published:** 2025-03-31

**Authors:** Shutaro Yamamoto, Yoshitaka Kinoshita, Haruki Kume, Takahiro Kimura, Takashi Yokoo, Eiji Kobayashi

**Affiliations:** 1 Department of Kidney Regenerative Medicine, The Jikei University School of Medicine, 3-25-8, Nishi-Shimbashi, Minato-ku, Tokyo 105-8461, Japan; 2 Department of Urology, The Jikei University School of Medicine, 3-25-8, Nishi-Shimbashi, Minato-ku, Tokyo 105-8461, Japan; 3 Division of Nephrology and Hypertension, Department of Internal Medicine, The Jikei University School of Medicine, 3-25-8, Nishi-Shimbashi, Minato-ku, Tokyo 105-8461, Japan; 4 Department of Urology, Graduate School of Medicine, The University of Tokyo, 7-3-1 Hongo, Bunkyo-ku, Tokyo 113-8654, Japan

**Keywords:** kidney transplant, sample transport, formalin

## Abstract

**Background:** Formalin is widely used as a standard fixative in histopathological analysis; however, its high toxicity and strict regulatory restrictions create challenges for the safe transport and external evaluation of specimens. In translational research utilizing large animal models, establishing a reliable transport protocol that preserves both tissue structure and antigenicity remains essential. **Objective:** This study aimed to develop and validate a protocol for the safe transport of formalin-fixed renal specimens while maintaining their histopathological and immunohistochemical integrity. **Methods:** Using a porcine model, renal specimens were fixed in formalin and subsequently substituted with physiological saline or 70% ethanol before transport. These were compared with specimens transported in formalin without substitution. Following transportation, hematoxylin and eosin (HE) staining and immunohistochemistry (Nephrin, E-cadherin, CD3) were performed to assess tissue integrity, antigenicity, and structural preservation. Additionally, sample degradation, antigen loss, and potential leakage were evaluated. **Results:** Specimens substituted with saline or ethanol retained cellular structure and antigenicity comparable to those transported in formalin, with no significant deterioration in histological or immunohistochemical quality. Furthermore, no leakage or sample damage was observed during transport, demonstrating the feasibility of this replacement protocol for routine pathological assessments. **Conclusions:** These findings suggest that formalin substitution with saline or ethanol provides a viable alternative for specimen transport, ensuring both biosafety and analytical integrity. This protocol may enhance specimen handling in preclinical research, regulatory compliance, and international collaboration in pathology and regenerative medicine.

## 1. Introduction

Formalin fixation has been a cornerstone of histological analysis for decades [1], particularly in the evaluation of laboratory animal specimens. In Japan, unlike human pathology, the evaluation of animal-derived specimens is limited to a few specialized centers capable of providing neutral and standardized pathological assessments. This limitation necessitates the transportation of formalin-fixed specimens to designated laboratories for subcontracted analysis. However, stringent regulations on the handling and transport of formalin in regions such as Japan, the US, and the EU raise significant legal and economic concerns regarding potential leaks during transit [2].

Porcine models have become essential tools in translational research due to their anatomical and physiological similarities to humans, particularly in regenerative medicine and pharmacological studies [3,4]. Recent advances, such as the transplantation of genetically modified porcine kidneys into humans, underscore the importance of these models in developing cutting-edge medical applications [5]. The demand for high-quality pathological analysis of porcine kidney samples continues to grow as these models provide insights unattainable in human clinical studies.

The need for a reliable protocol for transporting renal samples to specialized pathology centers is amplified by the increasing interest in xenotransplantation and kidney regeneration. Furthermore, the growing adoption of formalin-fixed paraffin-embedded (FFPE) samples in RNA sequencing and molecular analyses [6] necessitates transport methods that preserve specimen quality while adhering to safety standards.

Pathological evaluation relies not only on conventional histological assessment but also incorporates immunohistochemical analysis to detect specific cellular markers. Ensuring antigen preservation during transport is crucial for maintaining the accuracy of such evaluations. While formalin fixation effectively preserves tissue structures, its high toxicity and strict regulatory restrictions pose challenges for transportation. Therefore, replacing formalin with safer alternatives, such as physiological saline (0.9% NaCl) or ethanol, is a promising approach. However, it is essential to confirm that such substitutions do not compromise antigen integrity, ensuring reliable immunohistochemical assessments.

This study introduces a protocol specifically designed for transporting porcine kidney transplant samples to a designated laboratory for subcontracted analysis. The protocol ensures specimen integrity while addressing the risks associated with formalin handling by incorporating innovative steps such as replacing formalin with physiological saline or ethanol.

## 2. Materials and Methods

### 2.1. Experimental Animals

Six 4-month-old Large White × Landrace × Duroc (LWD) pigs, weighing 35–45 kg, were used to establish a renal transplantation model. All animals were euthanized after kidney sample collection. The experimental procedures were conducted at IVTeC Corporation’s laboratory (experiment numbers: M-23-022 and M-24-010) with approval from the Jikei University School of Medicine Ethics Committee (approval number: 2023-059) and the IVTeC Animal Experimentation Ethics Committee. All experiments complied with the Laboratory Animal Guidelines and management manuals.

### 2.2. Preparation for Remote Pathology Examination

#### 2.2.1. Kidney Sampling

**Figure 1:**
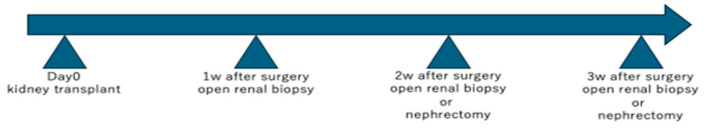
Experimental protocol of pig kidney transplant experiment.

Donor and recipient pigs (weighing 35–45 kg) were used in each experiment, with one pig serving as the donor and two as the recipients. Kidney transplant was performed as previously reported [7]. Kidney samples were obtained via wedge biopsies (5–10 mm^2^) or from entire removed kidneys postoperatively under open abdominal conditions, adhering to a predefined schedule (Figure 1).

Following kidney transplantation, the observation period was defined as up to 3 weeks. Wedge-shaped renal biopsies were performed laparotomically at weekly intervals, and nephrectomy was conducted at the time of euthanasia. The euthanasia schedule was determined according to the overall condition of each animal and the experimental plan.

#### 2.2.2. Formalin Fixation and Replacement Protocol

1. Initial Formalin Fixation

Tissue samples were immersed in neutral-buffered formalin at room temperature for 12–24 h, ensuring optimal fixation by mitigating the effects of blood and tissue interference.

2. Formalin Replacement

After initial fixation, the formalin solution was replaced with fresh neutral-buffered formalin to enhance fixation quality.

3. Refrigeration

Samples were refrigerated at 4 °C for 7 days post-fixation to maintain stability.

4. Physiological saline or Ethanol Replacement

Following fixation, formalin was replaced with either saline or 70% ethanol to prepare the samples for transport.

5. Transportation

The sample containers were filled with either saline solution or 70% ethanol. One sample was transported in formalin without the step of replacing the formalin with ethanol or saline. The samples were transported to the Sept.Sapie Pathology Analysis Centre (Mizuho-machi, Nishitama-gun, Tokyo, Japan) in refrigerated conditions. Transport took up to 24 h.

### 2.3. Pathological Analysis of Specimen

1. Histological Sample Preparation

Tissue specimens were promptly retrieved from physiological saline or 70% ethanol, sectioned, and transferred to neutral-buffered formalin for fixation. The specimens were dehydrated in a series of graded ethanol solutions according to standard protocols, embedded in paraffin, sectioned into 4 μm slices using a microtome, mounted on glass slides, and used for staining.

2. Hematoxylin and Eosin (HE) Staining

HE staining was performed following the standard histological protocols. All histological assessments were conducted at Sept. Sapie Co. LTD by a single board-certified pathologist to ensure consistency across all the samples. Additionally, selected images were captured using a fluorescence microscope (BZ-X800, Keyence, Osaka, Japan) at the Jikei University School of Medicine.

3. Immunohistochemistry for Nephrin, E-cadherin, CD3, and DAPI

For immunofluorescence staining, tissue sections were deparaffinized according to the standard histological protocols. Antigen retrieval was performed by autoclaving at 120 °C for 20 min. After blocking at room temperature for 1 h, the sections were washed three times with phosphate-buffered saline (PBS) and incubated overnight at 4 °C with the primary antibody (a list of primary antibodies is summarized in Table 1). The sections were then washed three times with PBS and incubated at room temperature for 1 h with Alexa Fluor 488-, 546-, and 647-conjugated secondary antibodies, along with DAPI for nuclear staining. Following three additional rinses in PBS, the sections were mounted using the SlowFade™ Diamond Antifade Mountant (Thermo Fisher Scientific, Waltham, MA, USA) and examined using a confocal fluorescence microscope (LSM980 confocal, Carl Zeiss, Oberkochen, Germany).

### 2.4. Statistical Analysis

A total of four images obtained from one tissue section per group were used for the analysis. The Kruskal–Wallis test was performed to compare the proportion of positively stained areas for Nephrin, ECAD, and DAPI among the three transport conditions (formalin, NS, EtOH). If a significant difference was detected (*p* < 0.05), pairwise comparisons were conducted using the Mann–Whitney U test with Dunn–Bonferroni correction to further investigate group differences. If no significant difference was found, the Mann–Whitney U test was still performed.

Additionally, to evaluate non-inferiority, the median difference and its 95% confidence interval (CI) between the formalin group and the alternative transport conditions were calculated. If the 95% CI included zero, the difference between groups was considered statistically non-significant, suggesting non-inferiority. Conversely, if the 95% CI did not include zero, a statistically significant difference was inferred. Due to varying degrees of rejection in the collected samples, the size of the CD3-positive area differed across specimens. Therefore, CD3 was excluded from this analysis. All statistical analyses were conducted using GraphPad Prism 10, with the significance level set at *p* < 0.05.

## 3. Results

### 3.1. Specimens Transfer

Samples transported using three different methods were obtained from two series of pig kidney transplantation experiments.

In one experiment, samples were obtained and transported in formalin or transported after fixation in formalin followed by substitution with physiological saline. In another experiment, all samples were fixed in formalin and subsequently substituted with 70% ethanol before transport.

Samples transported without substitution from formalin were handled directly by the authors in accordance with regulatory requirements, delivered to Sept. Sapie Co., and subsequently transferred to the facility using dedicated transport methods.

Samples substituted from formalin to saline or 70% ethanol were transported via a general freight company and delivered to the facility within 24 h over a distance of approximately 500 km, with no observed damage, including leakage.

### 3.2. HE Staining

**Figure 2:**
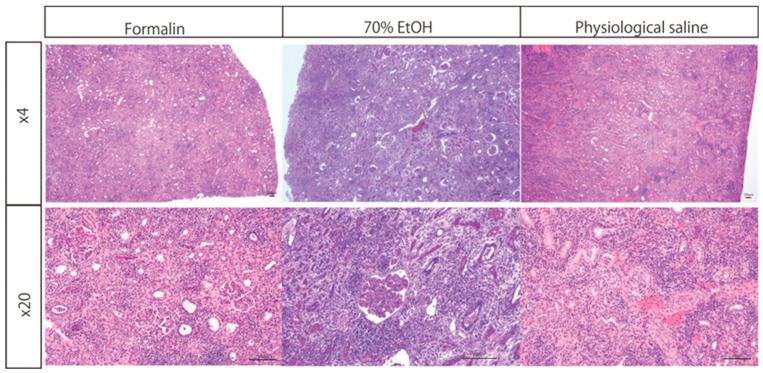
Histological evaluation of transplanted kidney tissue using HE staining under different post-fixation conditions.

Kidney specimens transported in formalin, saline, or ethanol were analyzed to evaluate their histological suitability. Inflammatory cell infiltration was observed across all groups, facilitating the assessment of rejection (Figure 2). Importantly, no structural or histological artifacts were identified in specimens subjected to saline or ethanol replacement prior to transport.

Kidney tissue samples were initially fixed in formalin and subsequently either transferred to 70% ethanol or saline or retained in formalin during transportation. HE staining was performed, and microscopic evaluation at 4× and 20× magnifications demonstrated well-preserved nuclear and cytoplasmic staining across all conditions. One sample per protocol was evaluated, and multiple images were captured per sample, although the exact number was not predetermined. The structural integrity of the transplanted kidney, including glomeruli, renal tubules, and infiltrating lymphocytes, was clearly maintained.

### 3.3. Immunohistochemistry

**Figure 3:**
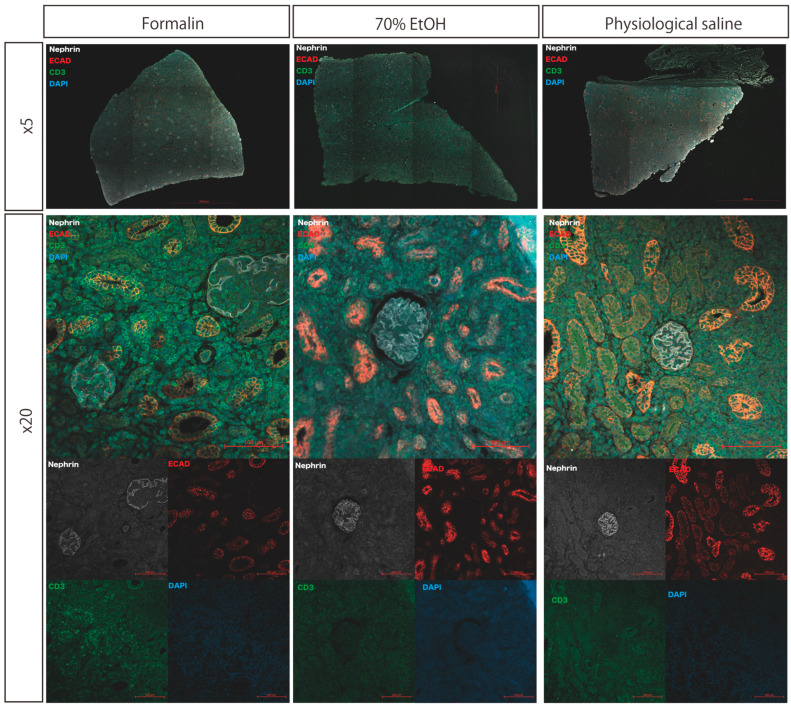
Immunofluorescence staining of transplanted kidney tissue under different transport conditions after formalin fixation.

Immunostaining was performed using Nephrin, E-cadherin, and CD3 as primary antibodies. Specific antigen staining was clearly observed for Nephrin (glomeruli), E-cadherin (renal tubules), and CD3 (T lymphocytes) in all tissue sections, regardless of the protocol. CD3-positive cells were consistently detected in the glomeruli, interstitial areas, and renal tubules. A qualitative comparison of staining intensity and quality showed no evident differences among the protocols (Figure 3).

Kidney tissue samples were initially fixed in formalin and subsequently either transferred to 70% ethanol or physiologic saline or maintained in formalin during transportation. Paraffin-embedded sections were subjected to immunofluorescence staining for Nephrin, E-cadherin (ECAD), CD3, and DAPI. Fluorescent signals for all target antigens were adequately retained across all conditions, allowing for the identification of glomeruli (Nephrin), renal tubules (ECAD), T lymphocytes (CD3), and cell nuclei (DAPI).

### 3.4. Statistical Analysis of Immunohistochemical Staining

**Figure 4:**
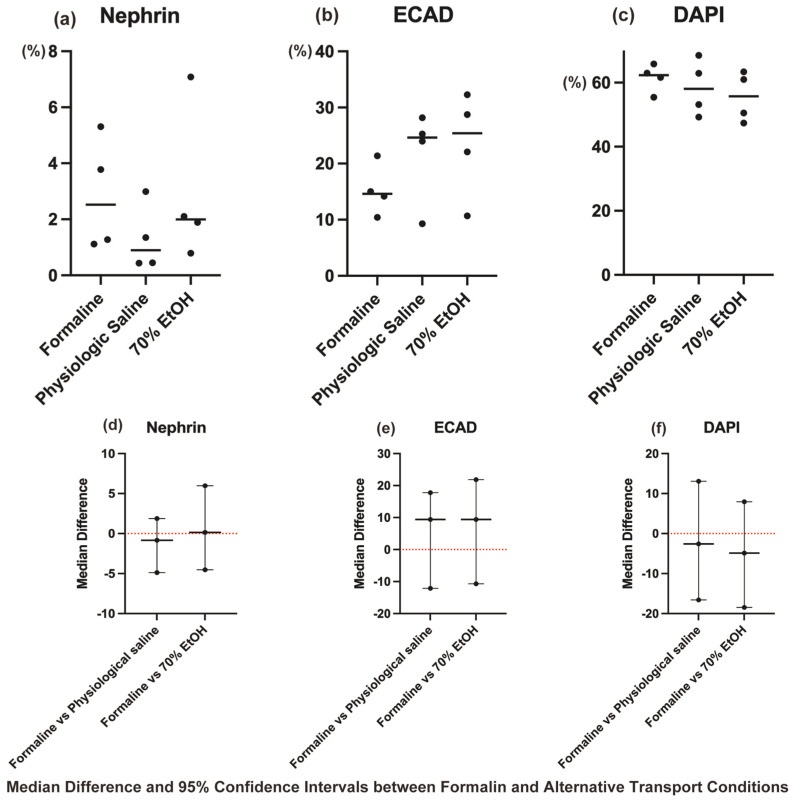
Comparison of immunohistochemical staining results and non-inferiority assessment. (**a**–**c**) Box plots showing the proportion of positively stained areas for Nephrin (**a**), E-cadherin (ECAD) (**b**), and DAPI (**c**) in different transport conditions (formalin, physiological saline, and ethanol). No statistically significant differences were observed among the groups (Kruskal–Wallis test: Nephrin, *p* = 0.4516; E-cadherin, *p* = 0.2958; DAPI, *p* = 0.5547). (**d**–**f**) Median differences and 95% confidence intervals (CIs) between formalin and the alternative transport conditions (physiological saline and ethanol) for Nephrin (**d**), ECAD (**e**), and DAPI (**f**), calculated using the Mann–Whitney U test. The red dashed line represents a median difference of 0. For all comparisons, the 95% CIs included zero, indicating no statistically significant differences and supporting the non-inferiority of the alternative transport conditions compared to formalin. In the Mann–Whitney U test, the *p*-values for Nephrin were 0.3429 for physiological saline vs. formalin and >0.9999 for 70% ethanol vs. formalin with 95% CIs of [−4.875, 1.869] and [−4.521, 5.967], respectively. The *p*-values for E-cadherin were 0.3429 for physiological saline vs. formalin and 0.2000 for 70% ethanol vs. formalin with 95% CIs of [−12.122, 17.757] and [−10.698, 21.864], respectively. For DAPI, the *p*-value was 0.6857 for physiological saline vs. formalin and 0.3429 for 70% ethanol vs. formalin with 95% CIs of [−16.576, 13.098] and [−18.460, 7.934], respectively.

The Kruskal–Wallis test revealed no statistically significant differences among the three transport conditions (formalin, physiological saline, and ethanol) for Nephrin, ECAD, and DAPI (*p* > 0.05, Figure 4a–c).

As no significant differences were detected, post hoc pairwise comparisons with Dunn–Bonferroni correction were not performed. However, to further evaluate non-inferiority, median differences and their 95% CIs were calculated for each protocol using the Mann–Whitney U test, with the formalin group as the reference (Figure 4d–f).

The results showed that for Nephrin, ECAD, and DAPI, all the 95% CIs included zero, supporting their comparability and suggesting non-inferiority to the formalin group.

These findings provide statistical evidence that physiological saline- and ethanol-substituted transport methods preserve antigen integrity at a level comparable to formalin-based transport, reinforcing their suitability as safer alternatives.

## 4. Discussion

In this study, we evaluated the effectiveness of a protocol for transporting pathological specimens using kidney samples. We demonstrated that replacing formalin with saline and 70% ethanol after fixation is an effective method to maintain specimen quality while improving transport safety. This protocol has the potential to serve as a standard for specimen transport in pathology and regenerative medicine, as it combines reduced leakage risk with enhanced regulatory compliance flexibility.

We confirmed that replacement with saline and 70% ethanol does not affect the structural stability or antigenicity of the specimens. Formalin has traditionally been used as a preservative that stabilizes cell structure and antigenicity [1]. Moreover, it has been reported that preservation fluids containing ethanol have a fixation capability comparable to that of formalin, while offering advantages in regulatory compliance and ease of handling [8,9]. In HE staining, cell structure was appropriately preserved, and antigenicity was maintained in immunostaining. Although ethanol-based preservation has been studied primarily in the context of fixation, its application for specimen transport has not been thoroughly explored. Additionally, our findings suggest that saline replacement provides a safer and more convenient alternative, reducing chemical exposure risks during transport while maintaining specimen integrity. These findings indicate that, in addition to improving transport safety, this protocol does not compromise the quality of pathological specimen analysis.

Compared to conventional formalin-fixed transport, this protocol enhances specimen reliability while minimizing leakage risks and ensuring regulatory compliance. To clearly delineate the advantages of our proposed protocol over conventional formalin-fixed transport, the key differences between the conventional formalin-fixed transport method and our proposed protocol are summarized in Table 2. This table outlines key improvements in biosafety, antigen preservation, and logistical feasibility, providing a structured assessment of the protocol’s practical benefits. Furthermore, it supports existing findings on both saline and ethanol-based preservation for antigen stability and extends these benefits to pathological specimen transport. This approach may strengthen the foundation of pathological analysis, facilitate international research collaboration, and contribute to the development of new therapeutic strategies. However, further validation is required in this specific field. In addition to its application in RNA sequencing, the potential for epigenome analysis using FFPE samples has been demonstrated in molecular biological research [10]. These findings suggest that this protocol may be applicable to RNA sequencing and other molecular analyses, although further validation is required to confirm RNA integrity under these conditions.

Despite these advantages, this study has some limitations. First, this study was restricted to kidney samples, and it remains unclear whether similar results hold true for other organs or tissues. It is necessary to evaluate the effectiveness of this method in organs with different tissue structures and preservation requirements, such as the liver and skin. Second, this study only considered short-term transport of up to 24 h, and the maintenance of quality during long-term storage was not assessed. To establish a more practical protocol, its applicability to extended storage periods should be examined. Third, the evaluation focused on tissue structure and protein antigenicity but did not verify DNA and RNA preservation for molecular biological analysis. Assessing molecular-level preservation will enable the development of protocols applicable beyond pathological analysis. Finally, this study was conducted on a small scale with a limited number of samples. Future research should focus on increasing the sample size to confirm the reproducibility of the findings.

Despite these limitations, this study demonstrates that improvements to the transport protocol for kidney samples contribute to safety, efficiency, and quality maintenance. In particular, replacing formalin with saline and 70% ethanol is crucial for regulatory compliance and reducing transport risks. This transport method is expected to be applicable to a wide range of fields, including regenerative medicine.

Future research should focus on refining the protocol and enhancing its reliability through further validation studies. Additionally, its applicability to other organs and tissues, long-term storage, and molecular biological analysis should be explored. The findings of this study represent an important step toward improving the efficiency and safety of pathological analysis, creating an environment where researchers can safely and effectively utilize specimens.

## Figures and Tables

**Table 1:** List of primary antibodies.

Antigen	Host	Supplier	Cat. No.	Dilution
Nephrin	Guinea pig	PROGEN	GP-N2	1:100
E-cadherin	Rabbit	Cell Signaling Technology	3195S	1:100
CD3	mouse	Agilent	M7254	1:100

**Table 2:** Summary of key differences between the conventional formalin-fixed transport method and our proposed protocol.

Feature	Conventional Formalin Transport	Proposed Saline/Ethanol Substitution Protocol
Fixation	Formalin fixation	Formalin fixation followed by substitution with saline/ethanol
Transport Medium	10% neutral-buffered formalin	0.9% saline or 70% ethanol
Safety Concerns	Toxicity, regulatory restrictions	Reduced toxicity, safer handling
Antigenicity Preservation	High (but potential antigen degradation over long transport)	Comparable antigenicity preservation
Transport Method	Specialized transport due to formalin restrictions	General freight transport

## Data Availability

All relevant data supporting the findings of this study are either included within the article or are available upon request from the corresponding author.

## Abbreviations

The following abbreviations are used in this manuscript:

CD3	Cluster of Differentiation 3
CI	confidence interval
DAPI	4′,6-diamidino-2-phenylindole
ECAD	E-cadherin
FFPE	formalin-fixed paraffin-embedded
HE	hematoxylin and eosin
LWD	White × Landrace × Duroc pigs
NaCl	sodium chloride
PBS	phosphate-buffered saline
